# JS‐K induces reactive oxygen species‐dependent anti‐cancer effects by targeting mitochondria respiratory chain complexes in gastric cancer

**DOI:** 10.1111/jcmm.14122

**Published:** 2019-01-22

**Authors:** Xudong Zhao, Aizhen Cai, Zheng Peng, Wenquan Liang, Hongqing Xi, Peiyu Li, Guozhu Chen, Jiyun Yu, Lin Chen

**Affiliations:** ^1^ Department of General Surgery Chinese People's Liberation Army General Hospital Beijing China; ^2^ Institute of Military Cognitive and Brain Sciences Academy of Military Medical Sciences Beijing China

**Keywords:** apoptosis, gastric cancer, JS‐K, mitochondria respire chain complex, nitric oxide, reactive oxygen species

## Abstract

As a nitric oxide (NO) donor prodrug, JS‐K inhibits cancer cell proliferation, induces the differentiation of human leukaemia cells, and triggers apoptotic cell death in various cancer models. However, the anti‐cancer effect of JS‐K in gastric cancer has not been reported. In this study, we found that JS‐K inhibited the proliferation of gastric cancer cells in vitro and in vivo and triggered mitochondrial apoptosis. Moreover, JS‐K induced a significant accumulation of reactive oxygen species (ROS), and the clearance of ROS by antioxidant reagents reversed JS‐K‐induced toxicity in gastric cancer cells and subcutaneous xenografts. Although JS‐K triggered significant NO release, NO scavenging had no effect on JS‐K‐induced toxicity in vivo and in vitro. Therefore, ROS, but not NO, mediated the anti‐cancer effects of JS‐K in gastric cancer. We also explored the potential mechanism of JS‐K‐induced ROS accumulation and found that JS‐K significantly down‐regulated the core proteins of mitochondria respiratory chain (MRC) complex I and IV, resulting in the reduction of MRC complex I and IV activity and the subsequent ROS production. Moreover, JS‐K inhibited the expression of antioxidant enzymes, including copper‐zinc‐containing superoxide dismutase (SOD1) and catalase, which contributed to the decrease of antioxidant enzymes activity and the subsequent inhibition of ROS clearance. Therefore, JS‐K may target MRC complex I and IV and antioxidant enzymes to exert ROS‐dependent anti‐cancer function, leading to the potential usage of JS‐K in the prevention and treatment of gastric cancer.

## 1 INTRODUCTION

As a lead anti‐cancer compound, O2‐(2,4‐dinitrophenyl) 1‐[(4‐ethoxycarbonyl) piperazin‐1‐yl] diazen‐1‐ium‐1,2‐diolate (JS‐K) exhibits significant anti‐cancer effects in many kinds of in vitro and in vivo cancer models.^1^, ^2^ JS‐K induces differentiation of human acute myeloid leukaemia HL‐60 cells,^3^ inhibits cell proliferation and triggers caspase‐dependent and caspase‐independent cell death in various cancer cell lines.^4^, ^5^, ^6^, ^7^, ^8^ Moreover, JS‐K also inhibits human cancer angiogenesis and metastasis^9^, ^10^ and enhances the cytotoxicity of some chemotherapeutic drugs, including cisplatin and arsenic, in drug‐resistant cells by increasing their intracellular drug concentration.^7^, ^11^ In addition, in the integrated animal, JS‐K significantly suppresses the growth of some cancer cells inoculated subcutaneously in mice, including human myeloid leukaemia cells,^3^ multiple myeloma cells,^6^ prostate cancer cells^3^ and non‐small‐cell lung cancer (NSCLC) cells,^12^ demonstrating that JS‐K is effective against both solid tumours and blood malignancies. However, it is not clear whether JS‐K is effective in killing gastric cancer cells.

As a nitric oxide (NO) donor prodrug, JS‐K is activated to release NO upon nucleophilic attack by reduced thiols, such as glutathione (GSH).^13^, ^14^ The reaction is catalysed by glutathione‐S‐transferases (GSTs), and JS‐K has been identified as a selective GSTα targeting compound.^3^, ^15^ Glutathione‐S‐transferases are phase II detoxification enzymes and catalyse the conjunction of xenobiotics with cellular reduced GSH; therefore, overexpression of GST in tumour cells allows tumour cells to gain a selective survival advantage over normal cells to chemotherapeutics by enhanced detoxification through GSH conjunction.^16^, ^17^ Therefore, up‐regulation of GST in tumour cells usually induces multi‐drug resistance, and GSTs have been regarded as potent targets for anti‐cancer drug design and synthesis.^17^, ^18^ The design strategy for JS‐K set out to exploit the overexpression of GST in malignant cancer cells compared with that in normal tissue. In line with this concept, JS‐K has been reported to selectively kill human multiple myeloma cells but not patient‐derived bone marrow stromal cells.^6^

Recently, JS‐K was reported to induce caspase‐dependent apoptosis by facilitating reactive oxygen species (ROS) accumulation,^5^, ^7^, ^12^, ^19^ but the specific target and detailed mechanism by which JS‐K triggers ROS accumulation is not completely understood. Reactive oxygen species are reactive chemical species containing oxygen and are formed as the by‐product of normal oxygen metabolism.^20^ The mitochondria respiratory chain (MRC) is the main source of the electrons required for ROS production; therefore, broken of MRC resulted from the inhibition of MRC complex activity promotes ROS production by facilitating electron escape from the MRC.^21^, ^22^ In normal cells, ROS are cleared by antioxidant enzymes, including superoxide dismutase (SOD), catalase and GSH peroxidase; therefore, the equilibrium between ROS production and clearance maintains ROS at low levels in normal cells.^21^, ^22^, ^23^, ^24^ However, suppression of the activity of MRC complexes and antioxidant enzymes promotes ROS production and inhibits ROS clearance, leading to ROS accumulation. High ROS levels usually cause damage to lipids, protein and DNA, and then induce oxidative stress or cell death; therefore, many kinds of chemotherapeutic drugs have been reported to kill cancer cells through facilitating ROS accumulation.^25^, ^26^

Gastric cancer is a leading cause of malignant death worldwide^27^; therefore, developing effective anti‐cancer reagents and exploring the molecular mechanisms of chemotherapeutics are fundamental for gastric cancer prevention and treatment. Therefore, the present study was designed to evaluate the effect of JS‐K on gastric cancer cells. We found that JS‐K inhibited gastric cancer cells proliferation by blocking the cell cycle in the G2‐M phase and suppressed the growth of gastric cancer subcutaneous xenografts. Although JS‐K induced significant ROS accumulation and NO release, the clearance of ROS, but not of NO, reversed JS‐K‐induced apoptosis and inhibition of gastric xenograft tumour growth; therefore, ROS accumulation is essential for the JS‐K‐induced toxic effect in gastric cancer. This mechanistic study on JS‐K‐induced ROS accumulation demonstrated that JS‐K significantly down‐regulated the core proteins of MRC complex I and IV, which contributed to the reduction of MRC complex I and IV activity and the subsequent ROS production. In addition, JS‐K also suppressed the expression of antioxidant enzymes, including copper‐zinc‐containing superoxide dismutase (SOD1) and catalase, resulting in the decrease of SOD1 and catalase activity and the subsequent inhibition of ROS clearance. Therefore, our study identified a potent target and molecular mechanism for JS‐K in mediating ROS‐dependent anti‐neoplastic effects in gastric cancer.

## 2 MATERIALS AND METHODS

### 2.1 Cells and reagents

The human gastric epithelial cell line GES‐1, and human gastric cancer cell lines, such as SGC7901, MGC803 and HGC27 cells, were obtained from the Cell Culture Centre, Beijing Institute of Basic Medical Science of the Chinese Academy of Medical Science (Beijing, China). The cells were cultured in RPMI Medium 1640 (Gibco, Grand Island, NY, USA) containing 10% foetal bovine serum (FBS, Kangyuan Biology, China). JS‐K was purchased from Santa Cruz Biotechnology (Santa Cruz, CA, USA) and dissolved in Dimethyl Sulfoxide (DMSO). Z‐VAD‐FMK (50 μmol/L), Z‐LEHD‐FMK (50 μmol/L) and Z‐DEVD‐FMK (50 μmol/L) were purchased from Medchem Express (Beijing, China). 3‐(4,5‐dimethyl‐2‐thiazolyl)‐2,5‐diphenyl‐2‐H‐tetrazolium bromide (MTT), carboxy‐PTIO (100 μmol/L) and NAC (500 μmol/L) were purchased from Sigma‐Aldrich (St. Louis, MO, USA).

### 2.2 Cell cycle analysis

Fifty thousand cells were fixed with 70% ethanol containing 1% FBS at −20°C overnight and then incubated with RNase A (20 µg/mL) at 37°C for 30 minutes, stained with propidium iodide (PI, 100 μg/mL) for 10 minutes, and then analysed by flow cytometry (FACS Calibur, BD, USA) and ModFit LT software (FACS Calibur). For each measurement, 10 000 cells were analysed, and the representative measurements were shown.

### 2.3 Cell growth assay

Cell growth was measured with an MTT assay. Briefly, cells were seeded into 96‐well plates (5 × 10^4^ cells per well). After 24 hours, cells were treated with or without JS‐K at the indicated concentrations for 48 hours, and then MTT was added into cell culture medium. DMSO was added to the wells to dilute the formazan in the cells after removing the culture medium, and then absorbances at OD 492 were determined. Cell survival ratios were calculated by normalizing the OD values of different groups with that of negative controls.

### 2.4 Clonogenic survival assay

One thousand SGC7901 cells were plated per well in 6‐well plates for 48 hours and followed by treatment with JS‐K at the indicated concentrations for another 48 hours. The cells were washed, fresh culture fluid was added, and colonies were stained with crystal violet 7 days later.

### 2.5 Apoptosis analysis

Cells were collected by trypsinization and stained with annexin V‐FITC and propidium iodide provided in the Apoptosis Assay Kit (Beyotime Institute of Biotechnology, Haimen, China), and then analysed with flow cytometry (FACS Calibur) and the Cell Quest software (FACS Calibur). More than 10 000 cells were analysed for each measurement. More than three independent experiments were performed in each group, and the representative measurements are shown.

### 2.6 Caspase activity assays

The activities of caspase 3 or caspase 9 were measured using the Caspase 3 or Caspase 9 Activity Assay Kits (Beyotime Institute of Biotechnology) according to the manufacturer's instruction. Briefly, cells were collected by trypsinization and lysed with lysis buffer. The protein concentrations in the lysates were quantified with a Bradford assay kit (Beyotime Institute of Biotechnology). The lysates were mixed with caspase 9 or caspase 3 substrates in a 96‐well plate and then incubated at 37°C for 30‐120 minutes. The absorbance was measured at 405 nm and used to calculate activities of caspase 9 or caspase 3. The relative caspase 9 or caspase 3 activities were calculated by normalizing the caspase 9 or caspase 3 activities in each group with those in the normal control group.

### 2.7 MMP measurement

MMP was measured with a Mitochondrial Membrane Potential Assay Kit and JC‐1 (Beyotime Institute of Biotechnology) according to the manufacturer's instructions. Briefly, 50 000 cells were collected by trypsinization and incubated with JC‐1 for 20 minutes at 37°C in the dark. The stained cells were washed twice with ice‐cold working solution and then analysed with flow cytometry (FACS Calibur) and the CELL Quest software (FACS Calibur). Twenty thousand cells were analysed for each measurement. JC‐1 aggregates in the polarized mitochondrial matrix and forms J‐aggregates, which emit red fluorescence at 595 nm when excited at 525 nm. However, JC‐1 cannot aggregate in depolarized mitochondrial matrices and, therefore, exists as JC‐1 monomers, which emit green fluorescence at 525 nm when excited at 485 nm. Mitochondria depolarization is indicated by a decrease in the red/green fluorescence intensity ratio.

### 2.8 Mitochondria isolation

Mitochondria were isolated with a Mitochondrial Isolation Kit (Applygen Technologies, Beijing, China). Fifty million cells were resuspended with ice‐cold Mito‐Cyto isolation buffer and homogenized with a grinder. The homogenate was centrifuged at 800 *g* for 10 minutes at 4°C. Supernatants were collected in a new tube and centrifuged at 10 000 *g* for 10 minutes at 4°C. The supernatant and pellet were saved as cytosolic and intact mitochondria fractions, respectively. The intact mitochondria were lysed with Laemmli Buffer (Bio‐Rad Laboratories, Hercules, CA, USA) to extract mitochondrial protein.

### 2.9 MRC complex activity measurements

Mitochondria respiratory chain complex activities were determined with Mitochondrial Respiratory Chain Complexes Activity Assay Kits (Genmed Scientifics, Shanghai, China). Briefly, the isolated mitochondria were resuspended with Mito‐Cito buffer (Applygen Technologies), frozen at −70°C and thawed at 37°C three times to extract the mitochondrial proteins. The protein concentration in the lysate was determined using a BCA Protein Assay Kit (Pierce, Rockford, IL, USA) and diluted to 0.1 μg/μL. The absorbance was determined on a Smartspec^TM^ Plus spectrophotometer (Bio‐Rad Laboratories). The MRC complex activities were detected by using a specific assay kit according to the manufacturer's instructions and calculated by normalizing the activities in different groups with those in the negative control group. All the measurements were performed in triplicate.

### 2.10 Gene silencing using small interfering RNA

SGC7901 cells were seeded in 6‐well plates for 24 hours, and then transfected with small interfering RNA (siRNA) against Cyto‐C (Genepharma, Shanghai, China) by using the Chemifect‐R (Fengrui Biology, Beijing, China) transfection reagents. The siRNA knockdown efficiency against Cyto‐C was evaluated by Western blot analysis. The siRNA target sequence against Cyto‐C is: 5ʹ‐actcttacacagccgccaata‐3ʹ.

### 2.11 Western blot analysis

For the Western blot experiments, cells and tissues were lysed in Laemmli buffer (Bio‐Rad Laboratories) and the protein concentration in the lysate was quantified with a BCA Protein Assay Kit (Pierce). Sixty micrograms of total protein were loaded in each lane, and then the proteins were separated by SDS‐PAGE and electrically transferred to a polyvinylidene difluoride membrane (Sigma‐Aldrich). After being blocked with 5% skim milk, the membrane was blotted with the appropriate primary antibodies for 12‐16 hours at 4°C and then incubated with the appropriate horseradish peroxidase‐conjugated secondary antibody (Zhongshan Biotechnology, Beijing, China) for 1‐2 hours at room temperature. Proteins were detected using the Tanon™ High‐sig ECL Western Blot Substrate (Tanon Science & Technology, Shanghai, China), and digital images were obtained using a Gel‐Imaging System (Tanon 5200, Shanghai, China). The following antibodies were used for the experiments: anti‐Ndufs4 (ab139178), anti‐catalase (ab16731) (Abcam biotechnology, Cambridge, MA, USA); anti‐Cyto‐c (sc‐13561), anti‐Cyto‐c oxidase subunit II (COX2) (sc‐514489) (Santa Cruz biotechnology); anti‐SOD1 (4266), anti‐VDAC (D73D12), anti‐Bcl‐2 (15071), anti‐Bcl‐xL(2764), anti‐PARP (9542), anti‐caspase 9 (9508), anti‐cleaved caspase 9 (9505), anti‐caspase 3 (9665), anti‐cleaved caspase 3 (9661) (Cell Signaling Technology, Beverly, MA, USA); anti‐GAPDH (G8795) and anti‐β‐actin (A5441) (Sigma‐Aldrich).

### 2.12 Ectopic expression of Bcl‐2 and Bcl‐xL

The plasmids expressing Bcl‐2 or Bcl‐xL and the empty negative control plasmid were purchased from Genechem (Shanghai, China). Plasmid transfections were performed using the Chemifect transfection reagent (Fengrui Biology) according to the manufacturer's protocol. Briefly, SGC7901 cells were seeded in 6‐well plates for 24 hours to reach 50%‐70% confluence, and then the transfection complex consisting of plasmid and Chemifect transfection reagent was added into the cell culture medium. After 48 hours, the ectopic expression efficiency was evaluated by Western blot.

### 2.13 ROS and NO measurements

Reactive oxygen species and NO were measured with a Reactive Oxygen Species Assay Kit and a NO Assay Kit (Beyotime Institute of Biotechnology), respectively. Briefly, cells were incubated with 5 μmol/L DCFH‐DA (for ROS measurement) or DAF‐FM DA (for NO measurement) for 30 minutes at 37°C in the dark and then measured by flow cytometry (FACS Calibur) at an excitation wavelength of 480 nm and an emission wavelength of 525 nm. Twenty thousand stained cells were analysed with flow cytometry for each measurement. The ROS and NO fold changes were calculated based on the mean geometry fluorescence determined with flow cytometry and shown as a histogram.

### 2.14 SOD1 and catalase activity measurements

The gastric cancer xenografts were lysed through homogenization, and then the protein lysate concentrations were quantified by using a BCA Protein Assay Kit (Pierce). The SOD1 activity in the gastric xenograft tumours lysates was measured by using the SOD1 Activity Assay Kit (Beyotime Institute of Biotechnology) according to the manufacturer's instructions. Briefly, the lysates were added to the detection buffer containing WST‐8 and xanthine oxidase, and then SOD1 suppresses the formation of WST‐8‐formazan produced from the reaction between WST‐8 and superoxide anion. The absorbance was measured at 450 nm, and the SOD1 activity was calculated based on the inhibition ratio of the WST‐8‐formazan production. The relative SOD1 activity was calculated by normalizing the SOD1 activity in each group with that in the normal control group. The catalase activity was measured by using a Catalase Activity Assay (Beyotime Institute of Biotechnology) according to the manufacturer's protocol. Briefly, the gastric xenograft tumour tissue lysates were added into a buffer containing hydrogen peroxide, and then incubated at 37°C for 45 minutes. Then, the catalase converted hydrogen peroxide into water and molecular oxygen. The remaining hydrogen peroxide was converted into N‐(4‐antipyryl)‐3‐chloro‐5‐sulfonate‐p‐benzoquinonemonoimine under the catalysis of the peroxidase, which can be detected by measuring the absorbance at 520 nm. The relative catalase activity was calculated by normalizing the catalase activity of all groups with that of the negative control group.

### 2.15 MDA measurement

Malondialdehyde content in gastric tumour tissue was measured by using an MDA Assay Kit (Beyotime Institute of Biotechnology) according to the manufacturer's instructions. Briefly, gastric cancer xenografts were lysed by homogenization and sonication, and then the lysates were added to a buffer containing thiobarbituric acid (TBA) which reacts with MDA to form the red adduct, MDA‐TBA. Absorbance at OD 535 was determined with a SmartspecTM Plus spectrophotometer (Bio‐Rad Laboratories), and the MDA content was calculated based on an MDA standard curve.

### 2.16 The gastric cancer subcutaneous xenograft study

The care and use of laboratory animals were in strict adherence with the NIH's guidelines. Meanwhile, all the animal experiments complied with the Chinese PLA General Hospital's Policy on the Care and Use of Laboratory Animals. Athymic BALB/c nude female mice (18‐22 g, 5‐6 weeks old) were purchased from Vital River Laboratories (Beijing, China). SGC7901 cells were harvested by trypsinization, washed with serum‐free 1640 medium, resuspended in phosphate buffered saline (PBS) and injected subcutaneously into the right flanks of mice (100 μL of PBS containing 5 × 10^6^ cells). When the tumours were palpable, the mice were randomly divided into three groups (six in each group) and administered JS‐K (1.5 and 3 mg/kg) via tail vein injection once every 2 days for a total 15 injections. The mice in the control group received an equal amount of normal saline. In addition, NAC (10 mg/kg) and carboxy‐PTIO (0.5 mg/kg) were administered by intraperitoneal injection. At the end of the study, the mice were killed, and the tumours were removed and weighed for statistical analysis.

### 2.17 Histopathology

Tissues were immediately collected from killed mice and fixed in 10% neutral buffered formalin for 48 hours. The fixed tissues were dehydrated in ethanol, cleared in xylene and embedded in paraffin blocks. Five‐micrometre sections were cut and mounted on adhesion microscope slides and then stained with haematoxylin and eosin (H&E) for analyses. Representative images were captured using identical settings in a Leica DM2500 optical microscope.

### 2.18 Activity measurement of ALT, AST and creatinine

Blood obtained from the mice was placed at room temperature for 60 minutes to clot and then centrifuged at 3000 *g* for 10 minutes at 4°C to isolate serum. The levels of AST, ALT and creatinine in serum were measured using specific assay kits (Jianglai biotechnology, Shanghai, China) according to the manufacturer's instructions in a microplate reader.

### 2.19 Statistical analysis

GraphPad Prism 7 software was used to analyse the data and construct statistical graphs. Statistical significance was analysed using ANOVA or unpaired *t* tests and defined as *P < 0.05 or **P < 0.01. All the experiments were repeated at least three times, and the data are expressed as the mean ± SD from representative experiments.

## 3 RESULTS

### 3.1 JS‐K inhibits proliferation of gastric cancer cells

**Figure 1:**
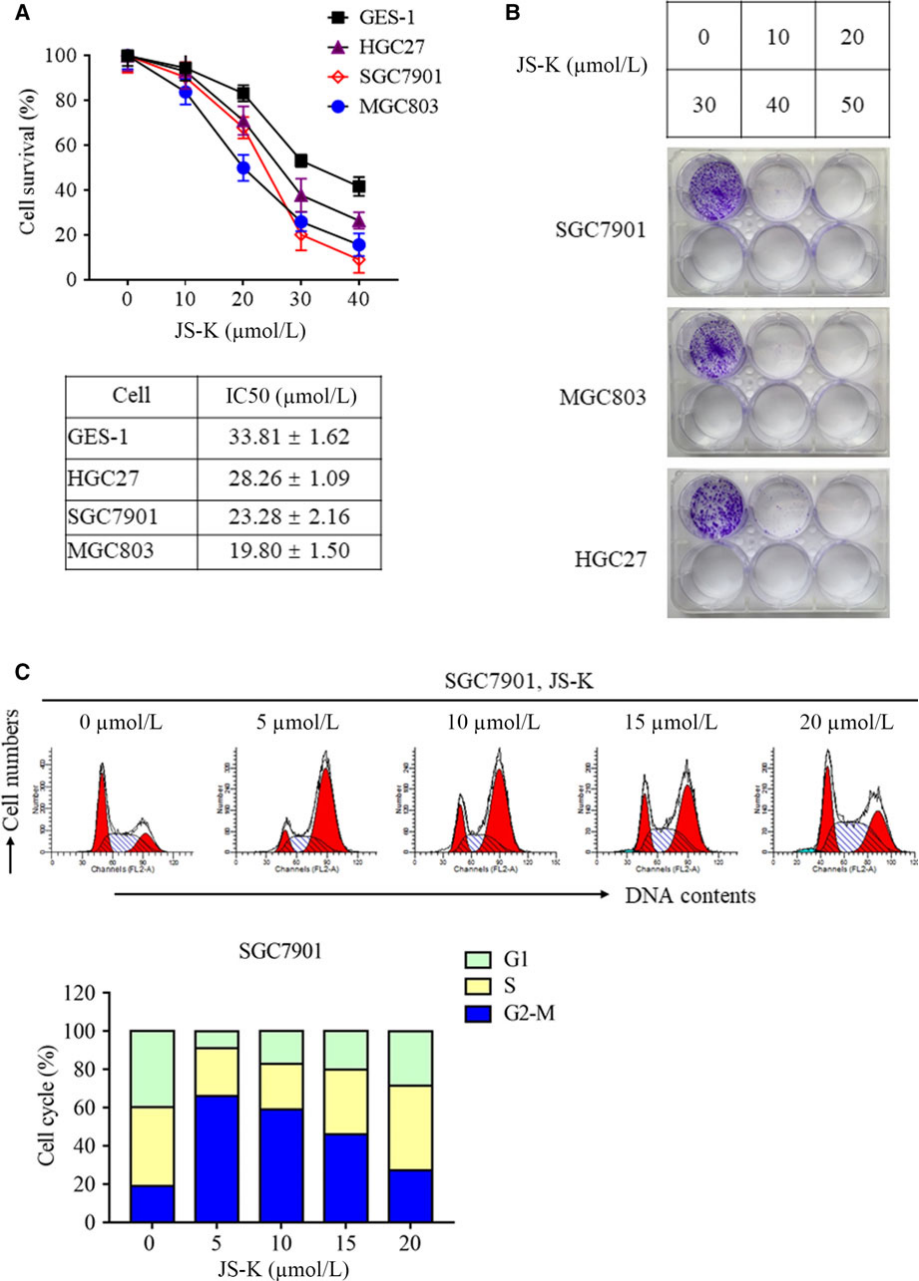
JS‐K inhibits cell proliferation in different gastric cancer cell lines. A, The effect of JS‐K on the proliferation of gastric epithelial cells and gastric cancer cells. GES‐1, SGC7901, MGC803 and HGC27 cells were treated with different JS‐K concentrations for 48 h, and an MTT assay was used to determine cell viability. Cell survival rates were calculated by normalizing cell survivals in different groups with those in the control group. The IC50 values were calculated by using the GraphPad Prism 7 software. B, JS‐K inhibits the clonogenic ability of different gastric cancer cell lines. SGC7901, MGC803 and HGC27 cells were treated with different JS‐K concentrations for 48 h and then cultured with medium without JS‐K for another 7 d. Cells were stained with crystal violet, and the representative plates of three independent experiments are shown. C, JS‐K induces G2‐M phase arrest in SGC7901 cells. Cells were treated with JS‐K at the indicated concentration for 12 h and then collected to determine the cell cycle phases with flow cytometry

Though JS‐K has been reported to suppress cell growth in different cancer models,^1^ its anti‐tumour effects in gastric cancer are not well understood. Therefore, we determined the effect of JS‐K on the proliferation of several kinds of gastric cancer cells. As shown in Figure 1A, JS‐K dose‐dependently decreased the survival of gastric cancer cell lines, including SGC7901, MGC803 and HGC27 cells, indicating that JS‐K suppressed gastric cancer cells proliferation. In addition, the 50% growth inhibition (IC50) of JS‐K in three gastric cancer cell lines was calculated based on the cell survival ratio data, and the IC50 values of JS‐K administered to SGC7901, MGC803 and HGC27 cells were 23.28, 19.80 and 28.26 μmol/L, respectively. MGC803 cells were the most sensitive to JS‐K‐induced cytotoxicity, and HGC27 cells were resistant to JS‐K‐induced cell growth arrest; therefore, we selected SGC7901 cells as a gastric cancer cell model for additional studies. In addition, the cytotoxic effect of JS‐K on the normal gastric epithelial cell GES‐1 was also determined. The results demonstrated that JS‐K also suppressed GES‐1 cell proliferation in a dose‐dependent manner (Figure 1A). However, the IC50 value of JS‐K for GES‐1 cells was 33.81 μmol/L, which is higher than that of gastric cancer cells, demonstrating that normal gastric epithelial cells are more resistant to JS‐K‐induced cytotoxicity. To further confirm the inhibitory effect of JS‐K on cell proliferation, we next determined the effect of JS‐K on clonogenic formation of different gastric cancer cell lines. As shown in Figure 1B, 10 μmol/L JS‐K significantly inhibited colony formation in SGC7901, MGC803 and HGC27 cells, further confirming the inhibitory effect of JS‐K against gastric cancer cell proliferation. Finally, we analysed the effect of JS‐K on cell cycle progression in SGC7901 cells, and the results demonstrated that JS‐K significantly induced G2‐M phase arrest in SGC7901 cells, contributing to JS‐K‐induced cell proliferation inhibition.

Collectively, JS‐K significantly suppressed gastric cancer cell proliferation, clonogenic formation and cell cycle progression.

### 3.2 JS‐K induces caspase‐dependent apoptosis in gastric cancer cells

**Figure 2:**
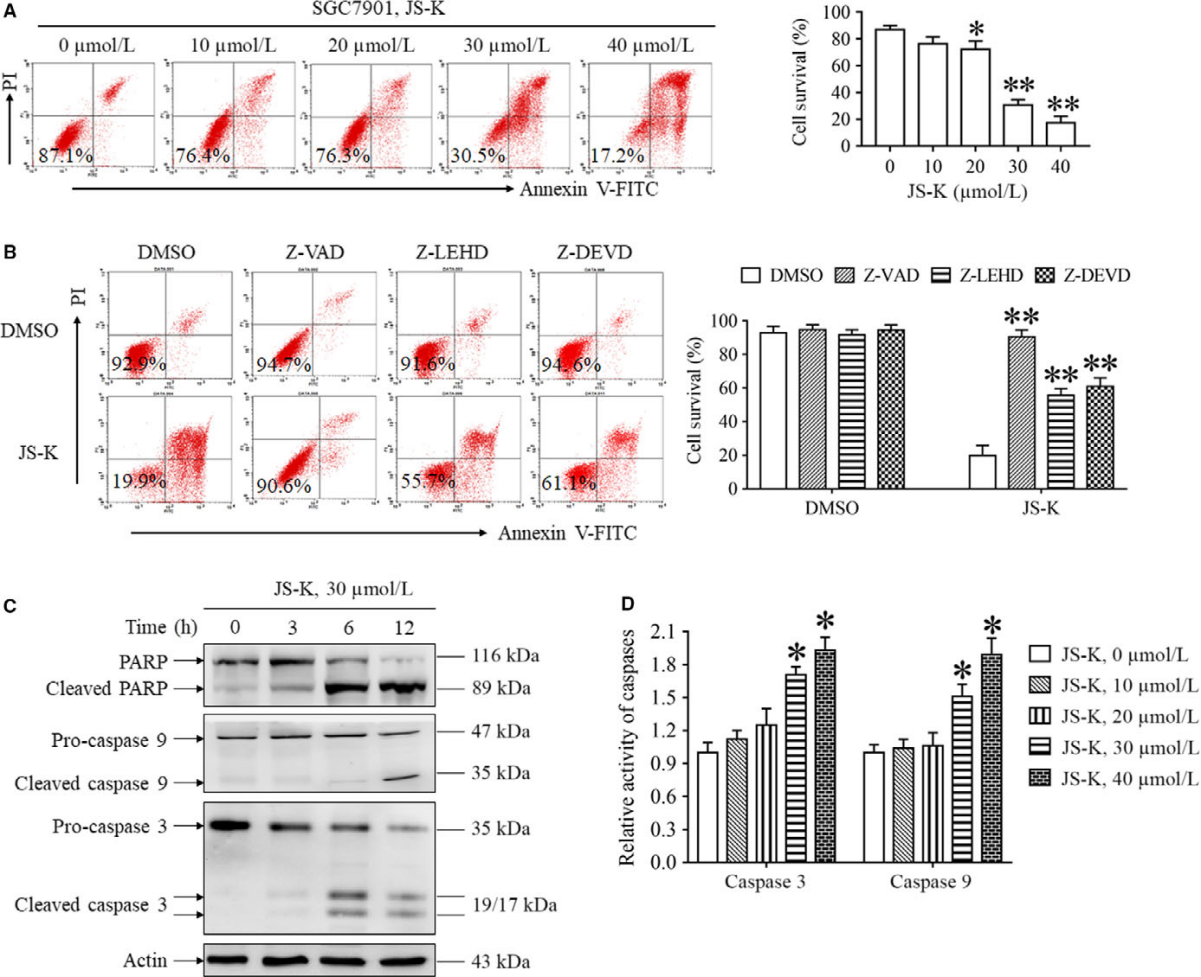
JS‐K induces caspase‐dependent apoptosis in SGC7901 cells. A, JS‐K induced apoptosis of SGC7901 cells in a dose‐dependent manner. Cells were treated with JS‐K at the indicated concentration for 24 h, and cell death was measured with flow cytometry. *P < 0.05. **P < 0.01. B, The effect of different caspase inhibitors on JS‐K‐induced cell death. SGC7901 cells were treated with JS‐K in the presence or absence of Z‐VAD (50 μmol/L), Z‐LEHD (50 μmol/L) or Z‐DEVD (50 μmol/L) for 24 h, and cell death was measured with flow cytometry. **P < 0.01. C. JS‐K induces PARP, caspase 9 and caspase 3 cleavage. SGC7901 cells were treated with JS‐K for the indicated time, and Western blotting was used to detect PARP, caspase 9 and caspase 3 cleavage. Actin was used as a loading control. D, JS‐K promotes caspase 9 and caspase 3 activation. SGC7901 cells were treated with JS‐K for 12 h and then harvested to measure the caspase 3 and caspase 9 activities with specific assay kits. More than three independent experiments were performed for each group, and the relative caspase activities were calculated by normalizing the caspase activities of all groups with the activities in a negative control group. *P < 0.05. **P < 0.01

JS‐K has been reported to induce caspase‐dependent and independent cell death in various cancer cell lines^4^, ^6^, ^8^, ^28^; therefore, we next detected whether JS‐K induces apoptosis in gastric cancer cell lines. As shown in Figure 2A,B, JS‐K induced SGC7901 cell death in a dose‐dependent manner, and Z‐VAD, a pan‐caspase inhibitor, almost completely inhibited JS‐K‐induced cell death, suggesting that JS‐K induced caspase‐dependent apoptosis. In addition, we found that JS‐K‐induced cell death was significantly inhibited by Z‐LEHD‐FMK and Z‐DEVD‐FMK, the caspase 9 and caspase 3 inhibitors, respectively, indicating that the activation of caspase 9/3 is essential for JS‐K‐induced apoptosis.

To further explore the mechanism of JS‐K‐induced cell death, we detected the effect of JS‐K on caspase signalling pathway activation in SGC7901 cells. As shown in Figure 2C, significant cleavage of caspase 9, caspase 3 and its substrate protein, poly (ADP‐ribose) polymerase (PARP), were detected in SGC7901 cells following JS‐K stimulation, indicating that JS‐K initiated caspase signalling pathway activation in gastric cancer cells. Moreover, we also found that the activities of caspase 9 and caspase 3 were significantly increased in SGC7901 cells following JS‐K administration (Figure 4D), further confirming the critical role of caspase signalling pathway activation in mediating JS‐K‐induced apoptosis.

Based on these findings, JS‐K induced caspase‐dependent apoptosis in gastric cancer cells.

### 3.3 ROS, but not NO, were essential for JS‐K‐induced gastric cancer cell death

**Figure 3:**
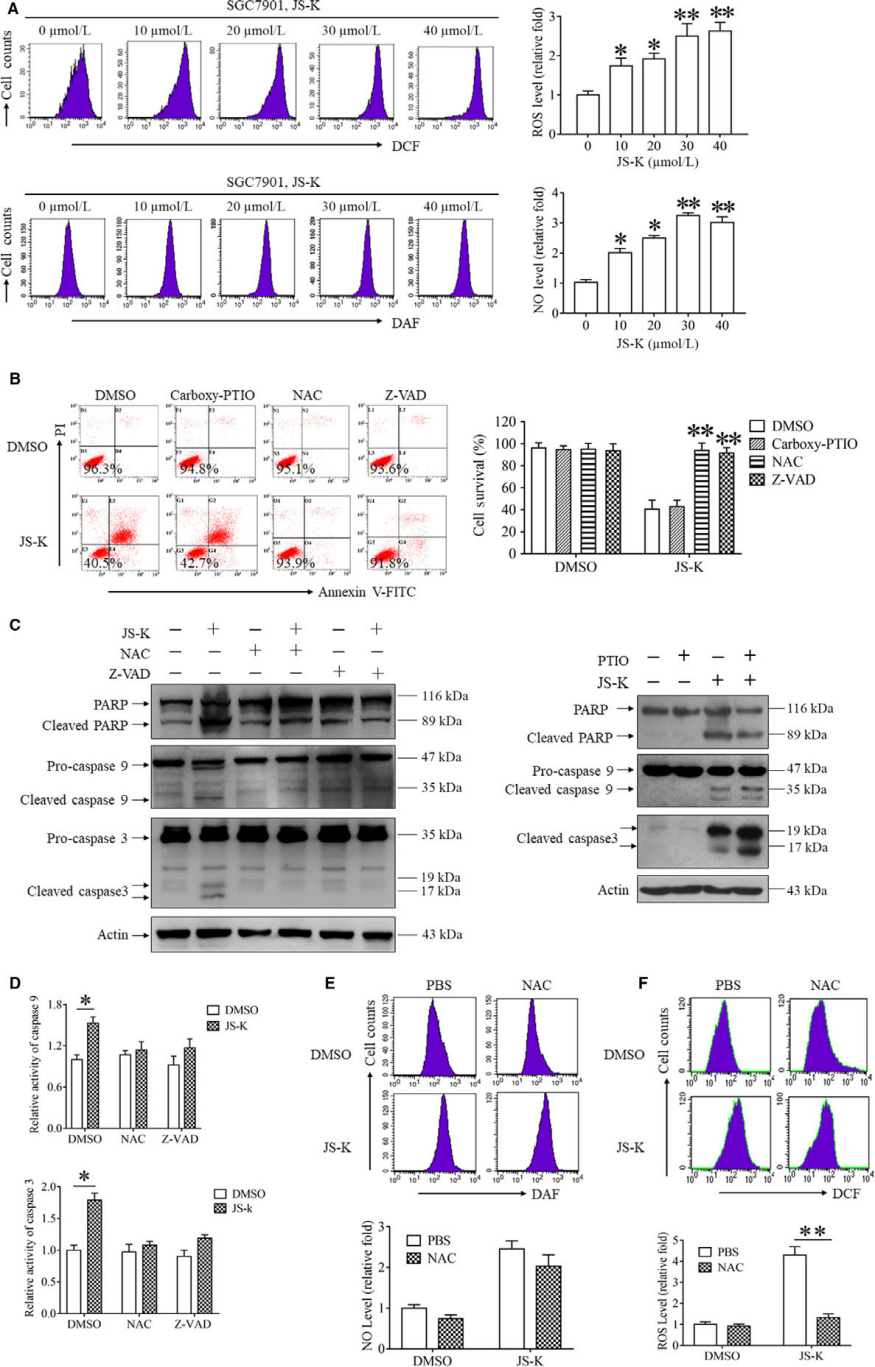
JS‐K‐induced cytotoxicity was mediated by reactive oxygen species (ROS) accumulation but not nitric oxide (NO) release. A, JS‐K induces ROS accumulation and NO release in a dose‐dependent manner. SGC7901 cells were treated with JS‐K at the indicated concentration for 3 h and then harvested to measure the ROS and NO levels with flow cytometry. Three independent experiments were performed for each group. The relative ROS or NO levels were calculated by normalizing the ROS or NO levels in all the groups with those in a control group. *P < 0.05. **P < 0.01. B, JS‐K‐induced cytotoxicity was reversed by a ROS clearance reagent but not a NO scavenger. SGC7901 cells were treated with JS‐K in the presence or absence of carboxy‐PTIO (100 μmol/L), N‐acetyl‐L‐cysteine (NAC) (500 μmol/L) and Z‐VAD (50 μmol/L) for 24 h, and cell survival was measured with flow cytometry. **P < 0.01. C, NAC, but not carboxy‐PTIO, inhibited the PARP, caspase 3 and caspase 9 cleavage induced by JS‐K. SGC7901 cells were treated with JS‐K in the presence or absence of NAC, carboxy‐PTIO or Z‐VAD for 12 h, and Western blot analysis was used to detect PARP, caspase 3 and caspase 9 cleavage. Actin was used as a loading control. D, NAC suppresses JS‐K‐induced caspase 3 and caspase 9 activation. SGC7901 cells were treated with JS‐K in the presence or absence of NAC or Z‐VAD for 12 h and then harvested to measure caspase 9 and caspase 3 activities with specific assay kits. The relative caspase activities were calculated by normalizing the caspase activities of all the groups with the activities of a negative control group. *P < 0.05. E, The effect of NAC on ROS accumulation and nitric oxide release induced by JS‐K. SGC7901 cells were treated with JS‐K in the presence or absence of NAC for 12 h and then harvested to measure the ROS or nitric oxide levels with flow cytometry. The relative ROS and nitric oxide levels were calculated by normalizing the level of ROS and nitric oxide in all the groups with those in a negative control group. **P < 0.01

JS‐K has been reported to trigger cell death by promoting ROS accumulation or NO release in different cancer cell lines^5^, ^6^, ^7^; therefore, we next determined the role of ROS and NO in mediating JS‐K‐induced gastric cancer cell death. First, we measured the levels of ROS and NO during the procession of JS‐K‐induced cell death. As shown in Figure 3A, JS‐K induced ROS and NO accumulation in a dose‐dependent manner in SGC7901 cells. However, JS‐K‐induced apoptosis was blocked by N‐acetyl‐L‐cysteine (NAC, ROS clearance reagent), but not carboxy‐PTIO (NO scavenger) (Figure 3B), indicating that the accumulation of ROS, but not NO, plays a critical role in mediating JS‐K‐induced gastric cancer cell death. Next, we determined the effect of ROS accumulation on caspase signalling pathway activation triggered by JS‐K. As shown in Figure 3C, similar to Z‐VAD, NAC significantly blocked the cleavage of PARP, caspase 3 and caspase 9 in SGC7901 cells following JS‐K stimulation, suggesting that ROS accumulation was essential for initiating caspase signalling pathway activation induced by JS‐K. In contrast to NAC, carboxy‐PTIO, the NO clearance reagent, had no significant effect on the cleavage of caspase 9, caspase 3 and its substrate PARP induced by JS‐K, indicating that NO was not involved in mediating caspase signalling pathway activation. Moreover, NAC also suppressed the JS‐K‐induced increase in caspase 9 and caspase 3 activity (Figure 3D), further confirming the indispensable role of ROS accumulation in JS‐K‐induced activation of caspase signalling pathways. In addition, we also determined the effect of NAC on ROS and NO levels during JS‐K‐induced apoptosis and found that NAC had no significant suppressive effect against NO release; however, NAC completely blocked ROS accumulation in SGC7901 cells following JS‐K treatment (Figure 3E,F), indicating that NAC reversed JS‐K‐induced cytotoxicity by clearing ROS but not NO.

Based on our data, JS‐K induced ROS and NO accumulation simultaneously in gastric cancer cells, but only ROS accumulation contributed to JS‐K‐induced cytotoxicity.

### 3.4 JS‐K induces cell death through activating the mitochondria apoptotic pathway

**Figure 4:**
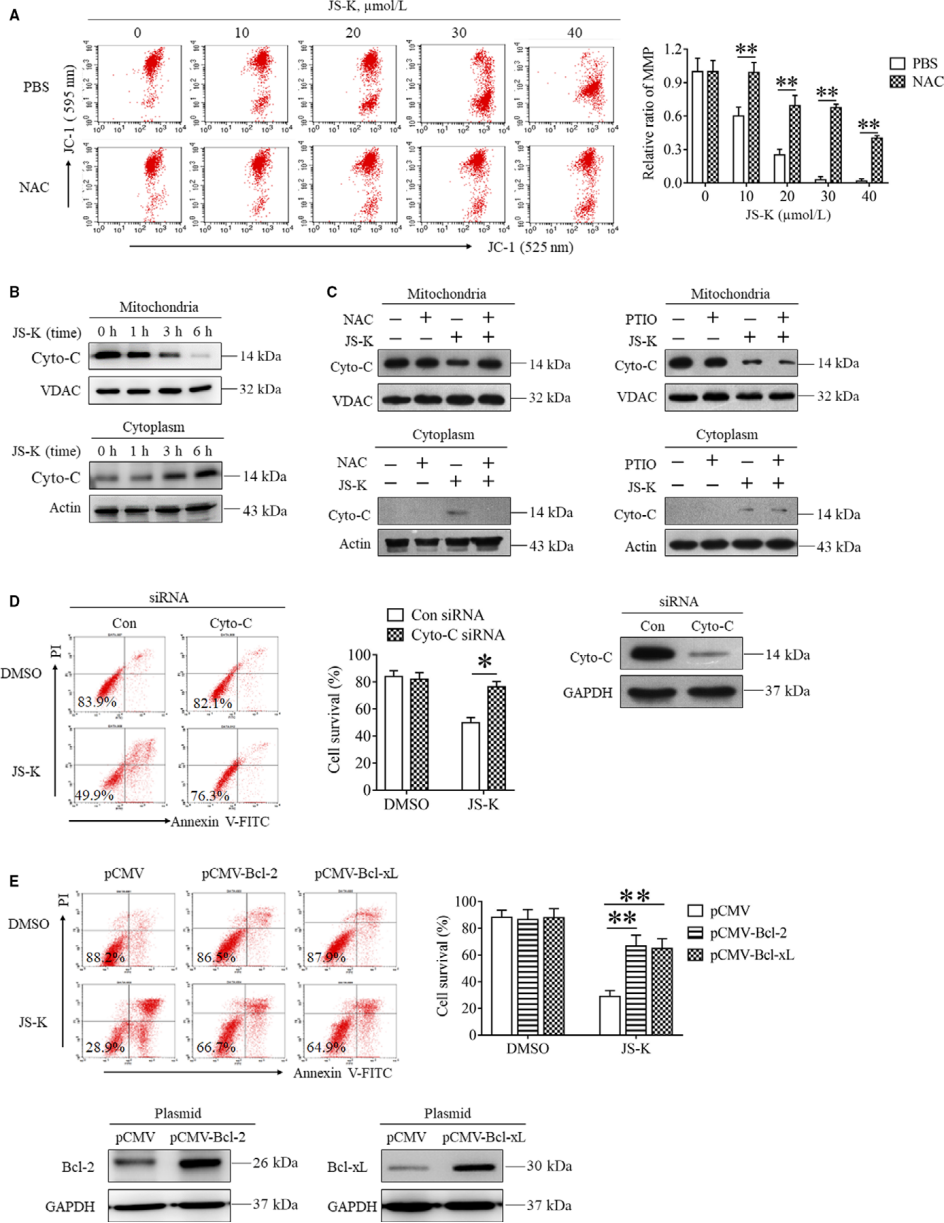
JS‐K induces reactive oxygen species‐dependent cytotoxicity by activating the mitochondria apoptosis pathway. A, NAC inhibits the depolarization of mitochondria induced by JS‐K. SGC7901 cells were treated with JS‐K in the presence or absence of NAC for 24 h, stained with JC‐1 and analysed with flow cytometry. The JC‐1 red/green fluorescence intensity ratio was normalized by comparing the data with the control group and is represented as relative mitochondrial membrane potential. Each experiment was performed in triplicate, and the representative measurements are shown. **P < 0.01. B, JS‐K induces the cytoplasmic release of Cytochrome c (Cyto‐C). SGC7901 cells were treated with 30 μmol/L JS‐K for the indicated time, and then harvested to isolate mitochondria and cytoplasm. Western blot analysis was used to detect the Cyto‐C levels in the mitochondria and cytoplasm. GAPDH and VADC were used as loading controls. C, The effect of NAC and carboxy‐PTIO on JS‐K‐induced Cyto‐C release. SGC7901 cells were treated with JS‐K in the presence or absence of NAC or carboxy‐PTIO for 12 h and then harvested to isolate mitochondria and cytoplasm. Western blot analysis was used to detect Cyto‐C levels in the mitochondria and cytoplasm. Actin and VADC were used as loading controls. D, The effect of Cyto‐C knockdown on JS‐K‐induced cell death. SGC7901 cells were transfected with Cyto‐C siRNA and a negative control siRNA for 24 h and then treated with or without JS‐K for another 24 h. Cells were stained with annexin V‐FITC and PI and analysed by flow cytometry. Western blot analysis was used to evaluate the efficiency of Cyto‐C knockdown. *P < 0.05. E, JS‐K‐induced apoptosis was inhibited by ectopic expression of Bcl‐2 or Bcl‐xL. SGC7901 cells were transfected with Bcl‐2, Bcl‐xL or negative control plasmids for 24 h, and then treated with 30 μmol/L JS‐K for another 24 h. Cells were stained with annexin V‐FITC and PI, and then analysed by flow cytometry. Western blot analysis was used to determine the Bcl‐2 and Bcl‐xL levels. GAPDH was used as a loading control. **P < 0.01

Reactive oxygen species have been shown to initiate apoptosis by activating the mitochondria apoptotic pathway^5^, ^7^; therefore, we first determined the effect of JS‐K on mitochondrial membrane potential (MMP). As shown in Figure 4A, JS‐K decreased the MMP of SGC7901 cells in a dose‐dependent manner, indicating that JS‐K induced mitochondria depolarization in gastric cancer cells. Moreover, NAC reversed the JS‐K‐induced decrease of MMP in SGC7901 cells (Figure 4A), suggesting that ROS played a critical role in mediating JS‐K‐induced mitochondria depolarization. Cytochrome c (Cyto‐C) usually releases from the depolarized mitochondria to the cytoplasm, and then activates caspase 9 to initiate apoptosis; therefore, we next detected the subcellular location of Cyto‐C in SGC7901 cells following JS‐K treatment. As shown in Figure 4B, JS‐K time‐dependently increased the Cyto‐C protein levels in the cytoplasm but decreased the Cyto‐C levels in the mitochondria, indicating that JS‐K induced cytoplasmic release of Cyto‐C. Moreover, JS‐K‐induced Cyto‐C release was reversed by NAC but not carboy‐PTIO (Figure 4B), further confirming the critical role of ROS, but not NO, in mediating the activation of the mitochondrial apoptotic pathway. Moreover, we found that Cyto‐C knockdown by using siRNA protected SGC7901 cells from JS‐K‐induced apoptosis (Figure 4D), indicating that the cytoplasmic releasing of Cyto‐C is essential for JS‐K‐triggered apoptosis. In addition, we found that ectopic expression of Bcl‐2 and Bcl‐xL, which are two proteins that protect against mitochondrial apoptosis, significantly protected SGC7901 cells from JS‐K‐induced cell death (Figure 4E), further confirming that JS‐K‐induced cell death occurs through mitochondria apoptotic pathway activation.

In conclusion, JS‐K induced ROS‐dependent mitochondria apoptotic cell death of gastric cancer cells by promoting mitochondria depolarization and subsequent cytoplasmic release of Cyto‐C.

### 3.5 In vivo anti‐tumour effects of JS‐K on gastric cancer cells

**Figure 5:**
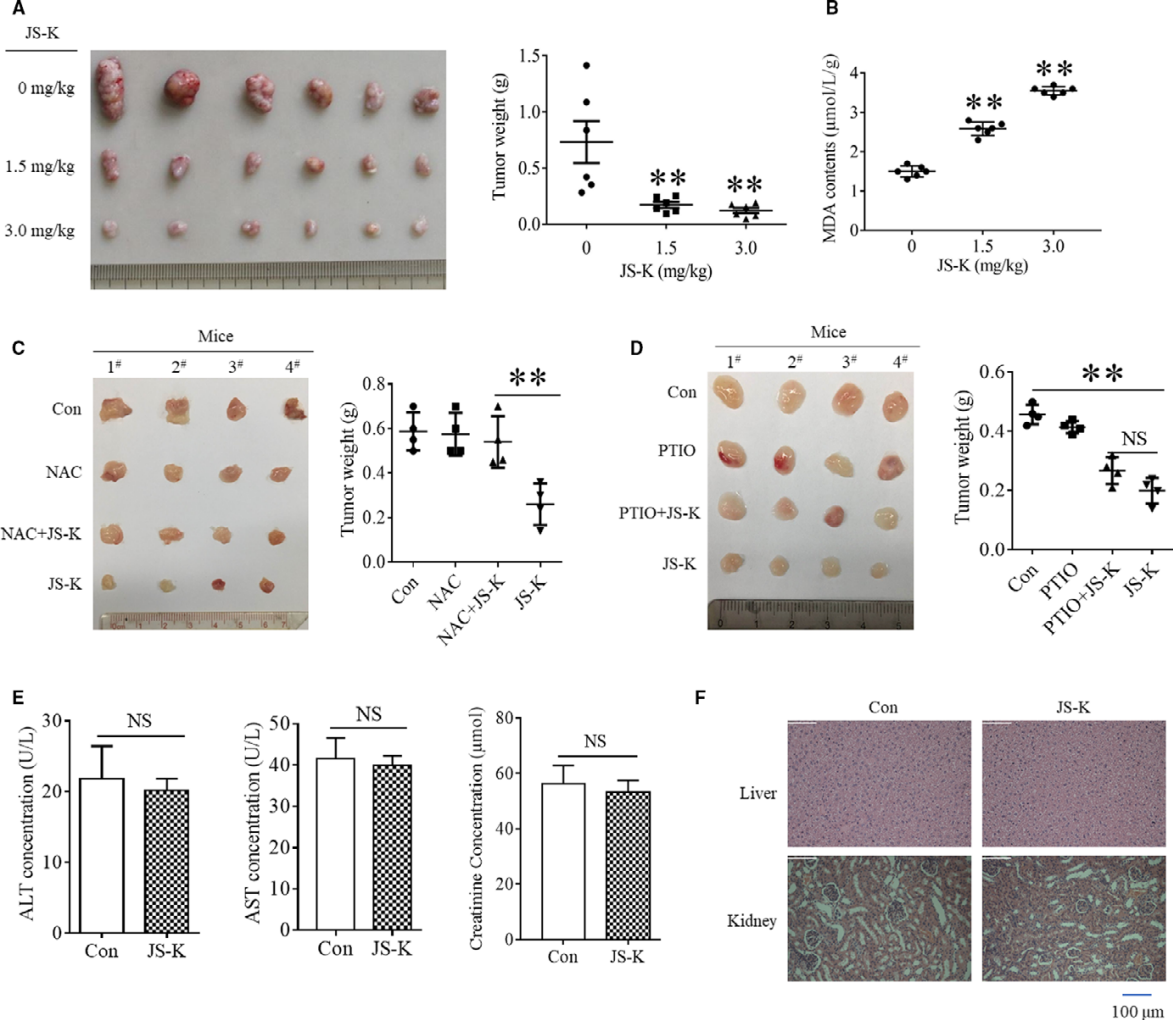
The anti‐tumour effects of JS‐K in a gastric cancer xenograft mouse model. BALB/c nude mice were injected subcutaneously with SGC7901 cells to establish the tumour‐bearing model, and JS‐K was administered once every 2 d. A, The in vivo anti‐tumour effect of JS‐K on gastric cancer. Tumour‐bearing mice were administered the indicated dose of JS‐K for 4 wk and then killed to isolate tumour tissue. The tumours were aligned according to size for imaging and then weighed using a microbalance. B, the malondialdehyde (MDA) content in the tumour tissues. The isolated tumour tissues were grinded and lysed, and the MDA level in the tumour tissue lysate was measured with a specific assay kit. **P < 0.01. (C,D) The effect of NAC and carboxy‐PTIO on JS‐K‐induced inhibition of gastric tumour growth. Tumour‐bearing mice were administered JS‐K (3 mg/kg) in the presence or absence of NAC (10 mg/kg) or carboxy‐PTIO (0.5 mg/kg) for 3 wk. The tumours were isolated from the killed mice, aligned according to size for imaging, and then weighed with a microbalance. **P < 0.01. (E,F) The effect of JS‐K on the liver and kidney. Mice were administered with JS‐K (3 mg/kg) once every 2 d for 30 d, and blood was obtained before being killed to isolate serum. The levels of alanine transaminase, aspartate transaminase and creatinine in serum were measured using specific assay kits. After blood collection, the mice were killed to isolate the liver and kidney. The tissues were cut into slices and then stained with haematoxylin and eosin. Four mice in each group were examined independently, and representative images are shown (×200)

As JS‐K has been identified to exhibit significant in vitro cytotoxic effects on gastric cancer cell lines, we next determined its in vivo anti‐tumour effects on gastric cancer cells. First, we established a gastric cancer mouse model by subcutaneously inoculating SGC7901 cells into the right flank region of nude mice. JS‐K was administered through the tail vein to evaluate its anti‐cancer effects. As shown in Figure 5A, the tumours were smaller in the JS‐K‐administered mice than those in the negative control group, indicating that JS‐K suppressed gastric cancer xenograft growth. Moreover, the average tumour weight was also lower in the JS‐K‐administered group than that in the negative group, further confirming the in vivo anti‐cancer effect of JS‐K against gastric cancer cells. As our data demonstrated that ROS accumulation contributed to JS‐K‐induced cytotoxicity in gastric cancer cells and that aberrant ROS usually induces oxidative stress in vivo, we next determined the oxidative state in a gastric cancer xenograft model. As malondialdehyde (MDA) is the product of lipid oxidation and is usually used to represent the oxidative stress level, we measured MDA levels in the gastric tumour tissues. As shown in Figure 5B, JS‐K administration increased MDA levels in the gastric cancer xenograft model in a dose‐dependent manner, which is consistent with the ROS accumulation in SGC7901 cells stimulated with JS‐K. In addition, the inhibitory effect of JS‐K on gastric tumour growth was almost completely reversed by the ROS clearance reagent NAC (Figure 5C), further confirming the critical role of ROS in mediating the anti‐cancer effect of JS‐K. However, the administration of carboxy‐PTIO had no significant effect on the gastric tumour growth inhibition induced by JS‐K (Figure 5D), further confirming that NO is not the main mediator of the anti‐cancer effects of JS‐K in gastric tumours. Finally, we determined the cytotoxicity of JS‐K on mouse livers and kidneys and found that serum activity of alanine transaminase (ALT), aspartate transaminase (AST) and creatinine showed no significant changes between the negative control mice and the JS‐K‐treated mice (Figure 5E), indicating that JS‐K had no significant cytotoxic effect on mouse livers and kidneys. In addition, no significant pathological changes were observed in liver and kidney tissues isolated from the negative control mice and the JS‐K‐treated mice (Figure 5F), confirming that JS‐K (3 mg/kg) had no significant toxic effect on mouse livers and kidneys.

Collectively, JS‐K significantly induced the growth inhibition of gastric cancer cells and oxidative stress in vivo.

### 3.6 JS‐K down‐regulates MRC complex I and IV core proteins, as well as the antioxidant enzymes

**Figure 6:**
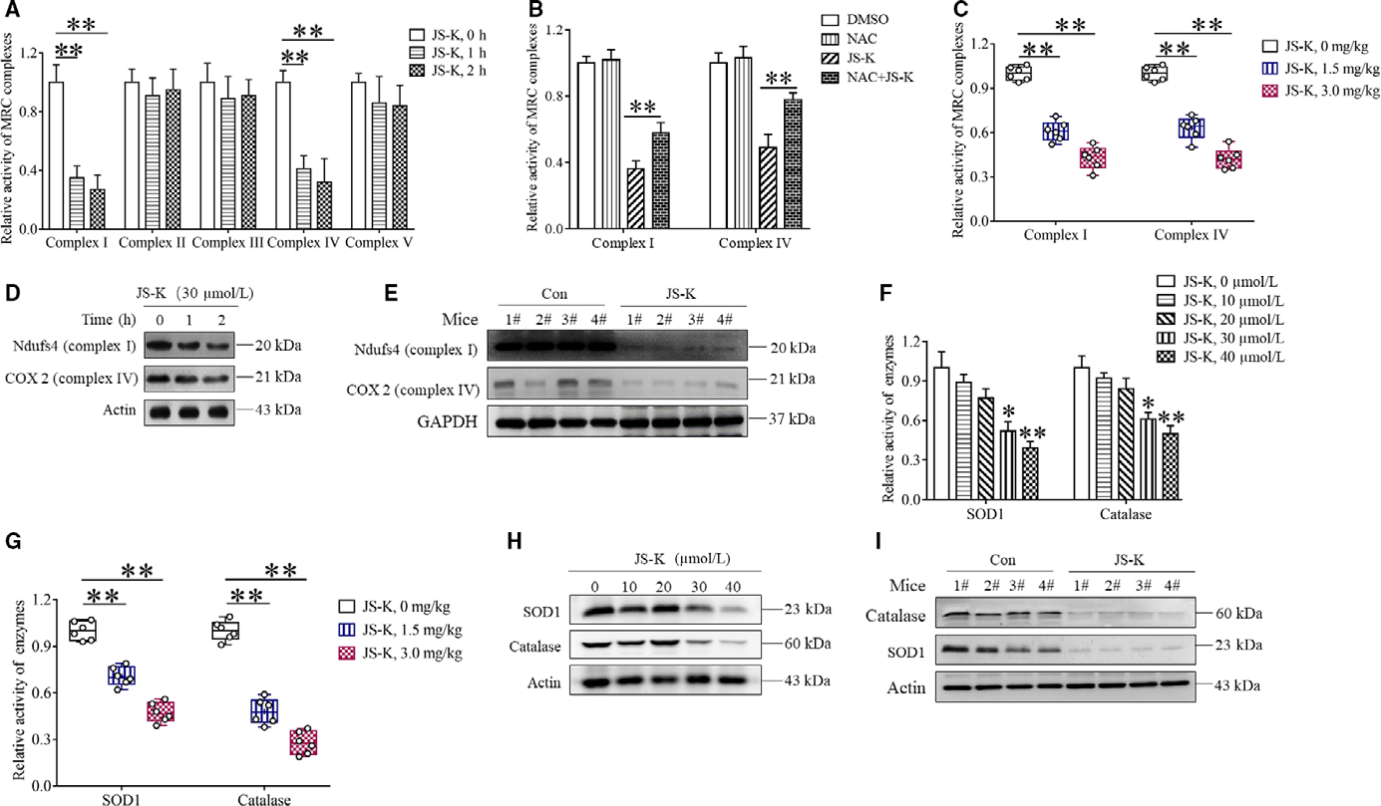
JS‐K down‐regulates mitochondria respiratory chain (MRC) complex I and IV core proteins, as well as antioxidant enzymes. A, The effect of JS‐K on MRC complex activities in SGC7901 cells. Cells were treated with JS‐K at the indicated concentration for 12 h and then harvested to measure MRC complex activities using specific assay kits. The relative activities of the MRC complexes were calculated by normalizing the MRC complex activities in all the groups with the activity of a negative control group. **P < 0.01. B, N‐acetyl‐L‐cysteine (NAC) partially reversed the reduction of MRC complex I and IV activity induced by JS‐K. SGC7901 cells were treated with JS‐K in the absence or presence of NAC for 12 h, and the MRC complex I and IV activity was measured using specific kits. **P < 0.01. C, JS‐K decreased MRC complex I and IV activity in gastric cancer xenografts. The isolated gastric tumour tissues were lysed by homogenization and sonication, and the activities of MRC complex I and IV in the lysates were measured with specific assay kits. The relative MRC complex activities were calculated by normalizing the activities of MRC complex I and IV in all the groups with those in a negative control group. **P < 0.01. D, JS‐K down‐regulates Ndufs4 and COX2 in SGC7901 cells. Cells were treated with JS‐K at the indicated time‐points and then collected to determine the protein level of Ndufs4 and COX2 by Western blot. Actin was used as a loading control. E, JS‐K administration suppresses Ndufs4 and COX2 expression in tumour tissues. The tumour tissues isolated from the mice used in Figure 5C were minced and lysed, and Western blot assays were used to determine the protein levels of Ndfus4 and COX2 in the tumour tissue lysates. GAPDH was used as a loading control. F, JS‐K decreases SOD1 and catalase activity in SGC7901 cells. Cells were treated with JS‐K at the indicated concentration for 12 h and collected to measure SOD1 and catalase activity using specific assay kits. The activity in JS‐K‐treated groups was normalized to that in the control group. *P < 0.05, **P < 0.01. G, JS‐K decreases SOD1 and catalase activity in gastric tumour tissues. The lysates of isolated gastric tumour tissues (Figure 5A) were used to measure the activities of SOD1 and catalase using specific assay kits, and the relative activities were normalized by comparing the activities of SOD1 and catalase in all groups with those in a negative control group. **P < 0.01. H, JS‐K down‐regulates SOD1 and catalase in SGC7901 cells. Cells were treated with JS‐K at the indicated concentrations for 12 h and then harvested to detect the protein levels of SOD1 and catalase with Western blot analysis. Actin was used as a loading control. I, JS‐K administration suppresses SOD1 and catalase expression in gastric tumour tissues. Tumour tissues were isolated from the negative control and JS‐K‐treated mice used in Figure 5C and minced and lysed to determine SOD1 and catalase protein levels using Western blot. Actin was used as a loading control

Although ROS accumulation mediated the anti‐cancer effect in gastric cancer cells in vitro and in vivo, the involved mechanism is not clear. Therefore, we next explored the mechanism for JS‐K‐induced ROS accumulation. Reduction of MRC complex activity promotes electron escape to produce ROS; therefore, we determined the effects of JS‐K on MRC complex activity. Figure 6A shows that JS‐K decreased the activities of MRC complex I and IV in a time‐dependent manner but had no inhibitory effect against MRC complex II, III and V in SGC7901 cells, indicating that the reduction of the MRC complex I and IV activities contributed to JS‐K‐induced ROS accumulation. As NAC partially reversed the negative effect of JS‐K on MRC complex I and IV activity (Figure 6B), it is possible that the accumulated ROS suppressed MRC complex I and IV activation in a feedback loop. In addition, we also detected the activities of MRC complex I and IV in gastric cancer xenografts and found that the activities of MRC complex I and IV were significantly lower in the JS‐K‐administered group than those in the negative control group (Figure 6C), further confirming that the reduction of MRC complex I and IV activity contribute to ROS accumulation induced by JS‐K. To explore the potential mechanism of JS‐K‐induced reduction of MRC complex I and IV activity, we determined the effect of JS‐K on the protein levels of Ndufs4 and COX2, the core enzymes in MRC complex I and IV, respectively. As shown in Figure 6D, JS‐K time‐dependently down‐regulated Ndufs4 and COX2 in SGC7901 cells. Moreover, JS‐K administration suppressed Ndufs4 and COX2 expression in gastric tumour tissues (Figure 6E). Therefore, JS‐K‐induced down‐regulation of MRC I and IV core proteins results in the reduction of MRC complex I and IV activity, and then promotes ROS production. In addition, ROS are usually cleared by antioxidant enzymes, including SOD1 and catalase; thus, the suppression of antioxidant enzyme activation also facilitated ROS accumulation. Therefore, we next explored the effect of JS‐K on SOD1 and catalase activity. As shown in Figure 6F, JS‐K induced a decrease in SOD1 and catalase enzyme activity in SGC7901 cells in a dose‐dependent manner. Moreover, JS‐K administration (1.5 and 3 mg/kg) significantly decreased SOD1 and catalase activity in gastric tumour tissues (Figure 6G); therefore, JS‐K could decrease SOD1 and catalase activity in vivo and in vitro, which would also contribute to JS‐K‐induced ROS accumulation. As SOD1 and catalase activity depends on their protein levels, we also determined whether JS‐K down‐regulated SOD1 and catalase expression. As shown in Figure 6H, JS‐K dose‐dependently decreased SOD1 and catalase protein levels in SGC7901 cells. Moreover, JS‐K administration also significantly suppressed the expression of SOD1 and catalase in gastric tumour tissues (Figure 6I), indicating that JS‐K down‐regulates SOD1 and catalase in vivo. Therefore, the down‐regulation of SOD1 and catalase induced by JS‐K may contribute to the reduction of SOD1 and catalase activity.

In summary, JS‐K induced ROS accumulation through down‐regulating MRC complex I and IV core proteins, as well as the antioxidant enzymes SOD1 and catalase.

## 4 DISCUSSION

As a promising anti‐cancer drug under development, JS‐K exhibits significant in vitro and in vivo anti‐cancer activity in many kinds of cancers,^1^, ^6^ but the effects of JS‐K in gastric cancer are unclear. In this study, we found that JS‐K inhibited gastric cancer cell proliferation by blocking the cell cycle at the G2‐M phase and suppressed gastric cancer xenograft growth in nude mice, demonstrating that JS‐K is effective against gastric cancer. In compliance with our results, JS‐K suppresses the growth of some solid tumour cells subcutaneously implanted into the flank of nude mice, including human prostate cancer (PPC‐1)^3^ and NSCLC (H1703 and H1944) cells.^12^ In addition, JS‐K also significantly inhibits the growth of HL‐60 and OPM1 multiple myeloma cells inoculated subcutaneously in mice.^3^, ^6^, ^29^ Therefore, JS‐K exhibits significant anti‐cancer activity in both solid tumours and blood malignancies. However, the role of JS‐K in the treatment of orthotopic tumours is controversial because JS‐K inhibited the growth of human hepatoma JM‐1 cells implanted intrahepatically in nude rats^2^; however, it did not result in tumour growth retardation or extend survival within tracranial U87 rat glioma.^30^ JS‐K has been reported to reduce the growth of U87 cells inoculated subcutaneously bilaterally into the flank of nude rats,^13^ and the dosage of JS‐K administration in the treatment of U87 cell subcutaneous xenografts (4 μmol/L/kg) is higher than that used in the treatment of orthotopic U87 rat glioma tumours (3.5 μmol/L/kg).^13^, ^30^ Therefore, low dosages of JS‐K administration may be not enough to suppress the growth of U87 orthotopic glioma. Moreover, the effective dosage of JS‐K administration used in the previous reports for the treatment of subcutaneous xenografts ranged from 4 to 75 μmol/L/kg,^3^, ^9^, ^12^, ^13^ and our results also demonstrate that 1.5 mg/kg (4 μmol/L/kg) JS‐K administration efficiently inhibited the growth of gastric cancer subcutaneous xenografts, further confirming that the failure of JS‐K treatment in orthotopic U87 rat glioma may be attributed to the low dosage of JS‐K that was administered. Therefore, our results and the previous studies all demonstrate that the appropriate dosage of JS‐K administration efficiently inhibits the growth of many kinds of cancer xenografts, including gastric cancer.

In this study, we found that JS‐K induced ROS accumulation in gastric cancer cells and oxidative stress in gastric tumour tissues. Moreover, NAC significantly reversed JS‐K‐induced ROS accumulation and the subsequent cytotoxicity. Therefore, ROS are essential for JS‐K to exert its anti‐tumour function in gastric cancer. In line with our observation, JS‐K also induces ROS accumulation in prostate, bladder and NSCLC cells, and the aberrant ROS trigger caspase‐dependent apoptosis by promoting mitochondrial depolarization and subsequent caspase pathway activation.^5^, ^7^, ^12^ Even in blood tumour cells, NAC protects leukaemia HL‐60 and U937 cells from JS‐K‐induced apoptosis.^4^, ^19^ Therefore, ROS accumulation plays a key role in mediating JS‐K‐induced cytotoxicity in solid tumours as well as blood malignancies. Reactive oxygen species accumulation usually leads to oxidative stress and DNA damage, which then activate c‐Jun N‐terminal kinase (JNK) and initiate apoptotic cell death.^26^, ^31^ Consistent with this concept, ROS accumulation and the subsequent JNK activation mediate JS‐K‐induced cell death and growth arrest in human hepatoma,^32^ NSCLC^33^ and multiple myeloma cells.^6^, ^34^ As many kinds of chemotherapeutic drugs, including arsenic trioxide and cisplatin, also exert their anti‐cancer activities by facilitating ROS accumulation,^11^ JS‐K is a promising anti‐cancer drug with a similar mechanism.

JS‐K is a NO donor prodrug, which releases high levels of NO upon enzymatic activation by GSTα.^1^, ^35^ As NO has been reported to induce apoptosis or differentiation in human leukaemia cells,^36^ it is possible for NO to mediate JS‐K‐induced cytotoxicity. In line with the postulation, JS‐K‐induced cytotoxic effects were abolished by cobalamin (Vitamin B12), a NO scavenger, in human multiple myeloma cells.^6^ Moreover, carboxy‐PITC, another NO scavenger, was found to reverse JS‐K‐induced growth inhibition of hepatoma carcinoma JM‐1 cells that were intrahepatically implanted into nude rats.^2^ Therefore, NO release has been identified as another important mechanism for JS‐K in killing tumours. In this study, we found that JS‐K significantly induced NO release and ROS accumulation simultaneously in gastric cancer cells; however, JS‐K‐induced cell death and tumour growth inhibition were blocked by NAC but not carboxy‐PTIO. In addition, we found that NAC almost completely suppressed ROS accumulation but had no effect on NO release. Therefore, high NO levels are not enough to initiate gastric cancer cell death in response to JS‐K stimulation, and ROS played a critical role in mediating the JS‐K‐induced anti‐cancer effects in gastric cancer cells. Consistent with our results, the NO scavenger, Vitamin 12, has no significant effect on the ability for JS‐K to kill the erythroleukemia SFFV‐MEL cells.^37^ Furthermore, low‐dose JS‐K induces cell death, NO release and ROS accumulation in sensitive NSCLC cells, but only NO release in resistant NSCLC cells.^12^ Therefore, it is easy to postulate that both NO release and ROS accumulation are required to mediate JS‐K‐induced cytotoxicity. Nitric oxide has been reported to be an antioxidant molecule that can be oxidized by ROS into nitrite and then trigger cell death in prostate and bladder cancer cells.^5^, ^7^, ^38^ Therefore, it is natural to hypothesis that NO release and ROS accumulation may work together to exert anti‐cancer effects in an integrated manner. Nitric oxide alone has been found to induce differentiation and apoptosis in human leukaemia cells^36^; therefore, we cannot exclude the possibility that NO mediates the anti‐cancer effects of JS‐K in some NO‐sensitive cancer cells in the absence of ROS accumulation.

Although ROS accumulation has been reported to mediate JS‐K‐induced anti‐cancer effects in different cancer cell lines,^4^, ^5^, ^7^, ^12^, ^19^ the target and involved mechanism for JS‐K in promoting ROS accumulation are not completely clear. Reactive oxygen species accumulation is usually derived from a disrupted balance between ROS production and clearance.^24^ The MRC is well known to be the main source of free electron, and inhibition of MRC complex activity leads to the MRC disruption, which then facilitates electron escape to form ROS.^39^, ^40^ Therefore, inhibition of MRC complex activation increases ROS production,^39^, ^40^ which has been identified as the main mechanism for ROS accumulation and ROS‐dependent cytotoxicity induced by some anti‐cancer compounds, including celastrol and rotenone.^41^, ^42^ In this study, we found that JS‐K induced a significant decrease in MRC complex I and IV activity in vitro and in vivo, which promoted ROS production and increased the cellular ROS levels. Therefore, MRC complex I and IV might be novel targets for JS‐K in mediating ROS accumulation. In addition, we also found that JS‐K decreased the activity of antioxidant enzymes, including SOD1 and catalase, which may facilitate ROS accumulation by inhibiting ROS clearance. Consistent with our results, JS‐K down‐regulated SOD1 expression in human leukaemia HL‐60 cells in a previous study.^43^ The disruption of reductase antioxidant systems, including SOD1, catalase or GSH peroxides, is well known to contribute to ROS‐dependent cytotoxicity for some chemotherapeutic agents.^24^, ^26^ Therefore, the suppression of antioxidant enzyme activation might be another mechanism by which JS‐K induces ROS accumulation. In addition, we also explored the potential mechanism for JS‐K‐induced reduction of MRC complex I and IV and antioxidant enzyme activity. We found that JS‐K significantly down‐regulated MRC complex I and IV core proteins and antioxidant enzymes SOD1 and catalase, which may result in the reduction of activity of MRC complex I and IV and antioxidant enzymes and the promotion of ROS accumulation. Therefore, suppressing the expression of MRC complex I and IV core proteins and antioxidant proteins may be the major mechanism for JS‐K to induce ROS accumulation. Furthermore, as a NO donor prodrug, JS‐K reacts with GSH to produce NO upon GSTα metabolism; therefore, JS‐K was identified to decrease cellular GSH levels in different cancer cells.^15^, ^19^ GSH depletion is known to facilitate ROS accumulation by suppressing ROS clearance; thus, we cannot exclude the possibility that JS‐K increases cellular ROS levels by inducing GSH depletion. Although these mechanisms might be cell type specific or selective, they are not mutually exclusive and work together to promote ROS accumulation induced by JS‐K in an integrated manner.

**Figure 7:**
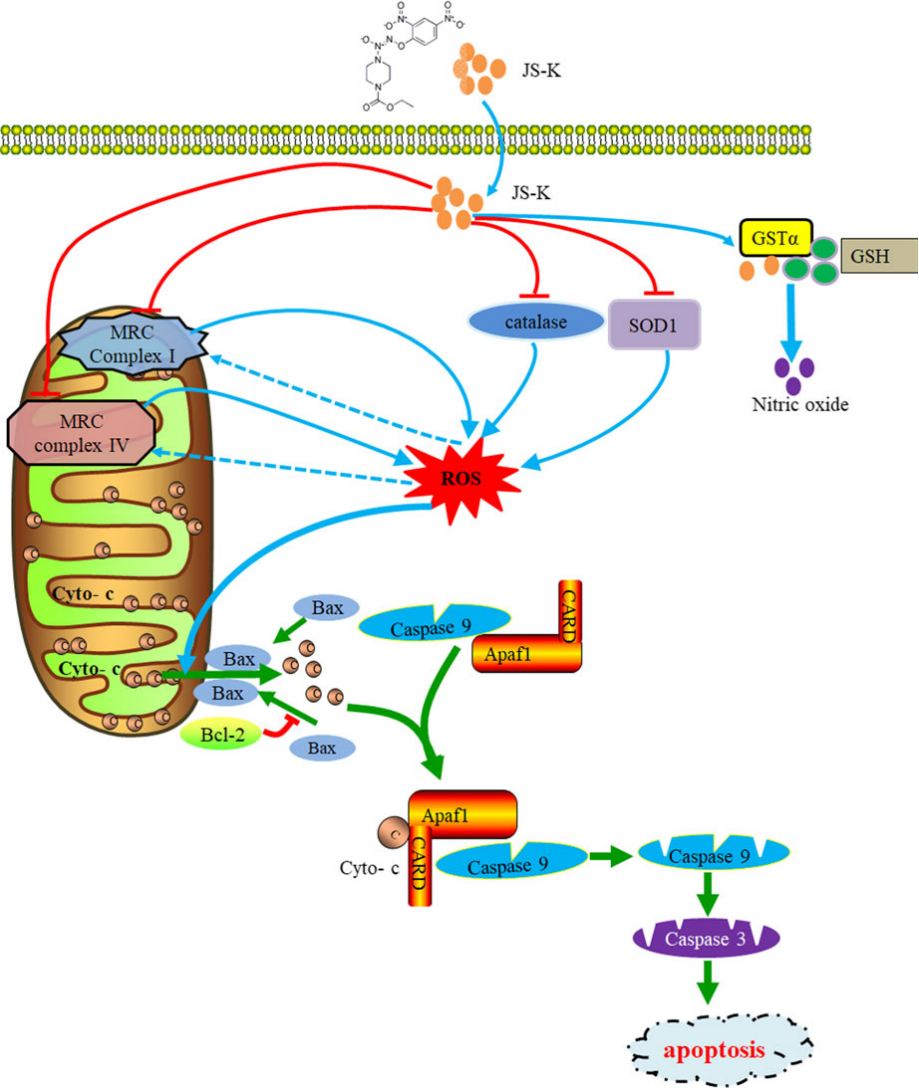
The model of JS‐K‐induced anti‐tumour activity in gastric cancer. As a lead anti‐cancer drug compound, JS‐K significantly suppressed the expression of the core proteins of mitochondria respiratory chain (MRC) complex I and IV, resulting in the reduction of MRC complex I and IV activity and the subsequent reactive oxygen species (ROS) production. In addition, JS‐K down‐regulated SOD1 and catalase, which facilitated the reduction of SOD1 and catalase reducing activity and promoted the inhibition of ROS clearance. The aberrant ROS then induces mitochondria depolarization, caspase signalling pathway activation and subsequent apoptotic cell death. Therefore, MRC complex I and IV or antioxidant enzymes act as novel targets for JS‐K in mediating ROS‐dependent anti‐cancer activity in gastric cancer

In summary, for the first time, we evaluated the anti‐cancer effects of JS‐K in gastric cancer in this study and found that JS‐K inhibited gastric cancer cell growth in vitro and in vivo. In addition, JS‐K induced the activation of the mitochondria apoptotic pathway and subsequent cell death by facilitating ROS accumulation but not NO release. We also explored the potential mechanism for JS‐K‐induced ROS accumulation and found that JS‐K significantly down‐regulated MRC complex I and IV core proteins and antioxidant enzymes, which resulted in the reduction of activity of MRC complex I and IV and antioxidant enzyme and then facilitated ROS production and clearance inhibition. These results raised the possibility that JS‐K targets the antioxidant enzymes and MRC complex I and IV to exert its anti‐tumour function (Figure 7). Therefore, our study determined the ROS‐dependent anti‐cancer effect of JS‐K in gastric cancer and identified novel targets and the involved mechanism for JS‐K‐induced ROS accumulation, leading to the development of JS‐K in cancer prevention and treatment.

## CONFLICTS OF INTEREST

The authors declare no conflicts of interest regarding this manuscript.

## AUTHOR CONTRIBUTIONS

Xudong Zhao, Aizhen Cai, Zheng Peng and Wenquan Liang finished all the experiments and analysed the data. Hongqing Xi and Peiyu Li prepared all the figures. Lin Chen, Jiyun Yu and Guozhu Chen wrote the paper. All the authors reviewed the manuscript.
